# Xanthogranulomatous Pyelonephritis: “Bear’s Paw Sign”

**DOI:** 10.5334/jbsr.1807

**Published:** 2019-05-13

**Authors:** Ju Ha Lee, Seung Soo Kim, Doo Sang Kim

**Affiliations:** 1 Soonchunhyang University College of Medicine, Cheonan Hospital, KR

**Keywords:** Xanthogranulomatous pyelonephritis, Kidney, Computed tomography

## Case History

**Figure 1:**
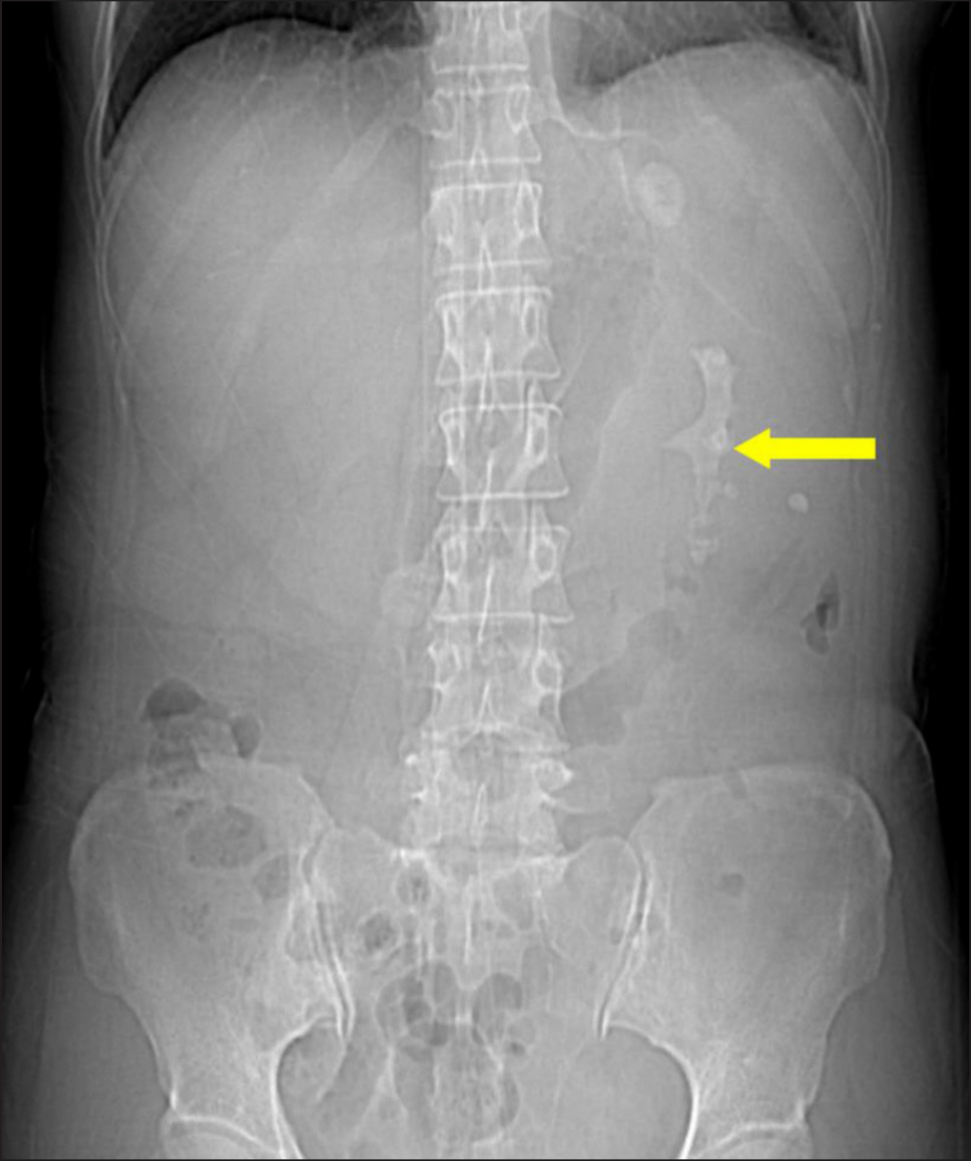
CT scanogram shows a staghorn calculus (arrow) in the location of the left kidney.

**Figure 2:**
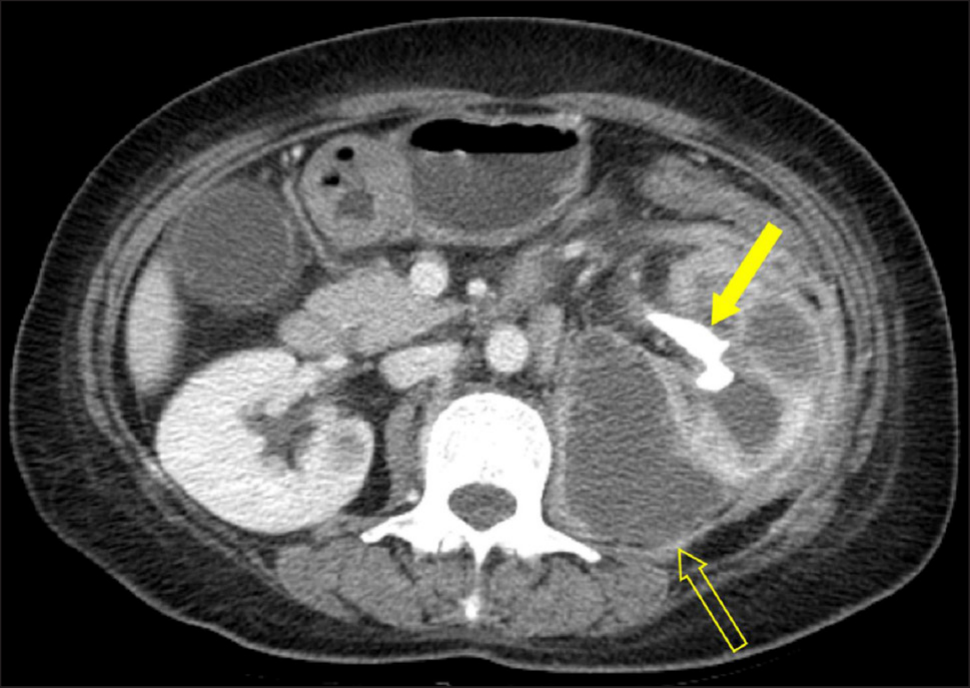
Axial contrast-enhanced CT image shows a calculus (arrow) within the contracted left renal pelvis and perirenal abscess (open arrow) involving the left psoas muscle.

**Figure 3:**
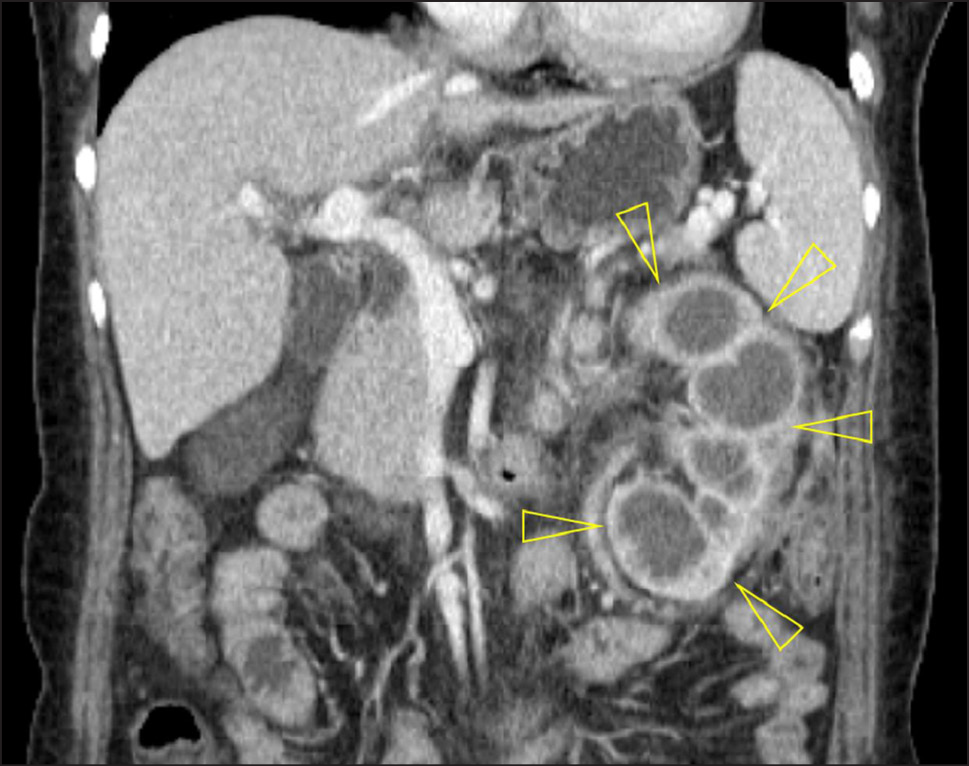
Coronal reformatted contrast-enhanced CT image shows an enlarged left kidney with surrounding inflammatory changes. The dilated renal calyces are similar to the footprint of a bear (open arrowheads).

A 55-year-old woman presenting with fever (up to 39°C) and left flank pain was admitted to our hospital. Serum levels of white blood cell count (14,630/mm^3^) and C-reactive protein (144.13 mg/L) were elevated. Urine analysis showed >100 leukocytes and 20–30 erythrocytes per high-power field. Contrast-enhanced computed tomography (CT) was performed under suspicion of acute pyelonephritis. The CT scanogram (Figure 1) showed staghorn calculus (arrow) in the left kidney. Axial contrast-enhanced CT image (Figure 2) showed an enlarged left kidney with surrounding fat infiltration, dilatation of the calices, a central calculus (arrow) within a contracted renal pelvis, and abscess (open arrow) around the left kidney. In coronal reformatted CT image (Figure 3), the left kidney looked like a footprint of bear (open arrowheads). The patient underwent nephrectomy and was diagnosed with xanthogranulomatous pyelonephritis (XGN).

## Comment

XGN is a granulomatous disease characterized by suppurative inflammation that leads to renal parenchymal destruction. An atypical immune response to chronic bacterial infection causes the renal parenchyma to be replaced by lipid-laden macrophages. Although most cases are associated with a renal pelvic calculus and consequent hydronephrosis, the renal parenchymal destruction results from diffuse inflammation rather than obstruction. XGN frequently develops in middle age and occurs twice more often in female patients than in males. Clinical symptoms of XGN are non-specific and vary: fever, dysuria, pyuria, leukocytosis, and flank pain. There are diffuse and focal types in XGN, and the diffuse form accounts for more than 90% of XGN [1].

XGN shows a typical image findings on contrast-enhanced CT including dilated renal calyces, renal calculus, inflammatory changes to an enlarged kidney, and abscess formation. Several hypoattenuating round areas throughout the enlarged kidney look like the pawprint of a bear, which are called “bear’s paw sign”. The hypoattenuating dilated calyces are not a collection fluid but a cellular infiltration of lipid-laden macrophages. A considerable number of patients present with perirenal abscess formation, which can involve adjacent organs and retroperitoneum [1]. Nephrectomy is the treatment of choice and XGN shows a high mortality rate if not treated.
